# Unmasking the enigma of lipid metabolism in metabolic dysfunction-associated steatotic liver disease: from mechanism to the clinic

**DOI:** 10.3389/fmed.2023.1294267

**Published:** 2023-11-27

**Authors:** Guocheng Rao, Xi Peng, Xinqiong Li, Kang An, He He, Xianghui Fu, Shuangqing Li, Zhenmei An

**Affiliations:** ^1^Department of Endocrinology and Metabolism, West China Hospital, Sichuan University, Chengdu, China; ^2^Department of Endocrinology and Metabolism, Affiliated Hospital of North Sichuan Medical College, North Sichuan Medical College, Nanchong, China; ^3^State Key Laboratory of Biotherapy and Cancer Center, West China Hospital, Sichuan University and Collaborative Innovation Center of Biotherapy, Chengdu, China; ^4^General Practice Ward/International Medical Center Ward, General Practice Medical Center, National Clinical Research Center for Geriatrics, Multimorbidity Laboratory, West China Hospital, Sichuan University, Chengdu, China; ^5^Department of Laboratory Medicine, West China Hospital, Sichuan University, Chengdu, China

**Keywords:** metabolic dysfunction-associated steatotic liver disease, lipid metabolism, *de novo* lipogenesis, drug intervention, nonalcohol fatty liver disease

## Abstract

Metabolic dysfunction-associated steatotic liver disease (MASLD), formerly defined as non-alcoholic fatty liver disease (NAFLD), is a disorder marked by the excessive deposition of lipids in the liver, giving rise to a spectrum of liver pathologies encompassing steatohepatitis, fibrosis/cirrhosis, and hepatocellular carcinoma. Despite the alarming increase in its prevalence, the US Food and Drug Administration has yet to approve effective pharmacological therapeutics for clinical use. MASLD is characterized by the accretion of lipids within the hepatic system, arising from a disarray in lipid provision (whether through the absorption of circulating lipids or *de novo* lipogenesis) and lipid elimination (via free fatty acid oxidation or the secretion of triglyceride-rich lipoproteins). This disarray leads to the accumulation of lipotoxic substances, cellular pressure, damage, and fibrosis. Indeed, the regulation of the lipid metabolism pathway is intricate and multifaceted, involving a myriad of factors, such as membrane transport proteins, metabolic enzymes, and transcription factors. Here, we will review the existing literature on the key process of lipid metabolism in MASLD to understand the latest progress in this molecular mechanism. Notably, *de novo* lipogenesis and the roles of its two main transcription factors and other key metabolic enzymes are highlighted. Furthermore, we will delve into the realm of drug research, examining the recent progress made in understanding lipid metabolism in MASLD. Additionally, we will outline prospective avenues for future drug research on MASLD based on our unique perspectives.

## Introduction

Metabolic dysfunction-associated steatotic liver disease (MASLD), previously known as non-alcoholic fatty liver disease (NAFLD), is a significant and rapidly increasing health concern in developed nations, becoming a leading cause of liver-related mortality. Its prevalence is also soaring in developing regions, highlighting the global impact of this condition (1). Albeit the alteration in nomenclature, the discernments pertaining to NAFLD remain pertinent to MASLD (2). In 2020, the epidemiological inquiry revealed that approximately 1.7 billion individuals worldwide have MASLD (3), and it is estimated that by 2030, approximately one third of the global population will be impacted by this disease (4). The prevalence of MASLD in the general population is around 6.3%–33% (5), 65% in obese people, 55.5% in people with diabetes, and as high as 72% in patients with dyslipidemia (4, 6, 7). Notably, the prevalence of MASLD tends to increase with advancing age, with less than 20% of cases observed in individuals below 20 years old and over 40% observed in those above 60 years old. Older patients diagnosed with MASLD exhibit a heightened vulnerability to liver fibrosis and cirrhosis (8). Furthermore, males exhibit a higher propensity for progression to metabolic dysfunction-associated steatohepatitis (MASH) and cirrhosis compared to females (8). In China, the rate of MASLD prevalence is escalating at an alarming pace, reaching more than twice that of western countries. In the general population of China, the prevalence of MASLD is close to 30%, which is the primary cause of chronic liver disease, and there are also differences among individuals in different provinces and regions, age groups, sexes, and metabolic situations (9). In the past two decades, the high prevalence of MASLD, along with the associated mortality rates for liver and extrahepatic disorders, has posed significant threats to human well-being, imposing substantial social and economic burdens on affected families and nations (10).

## Overview of MASLD

**Figure 1:**
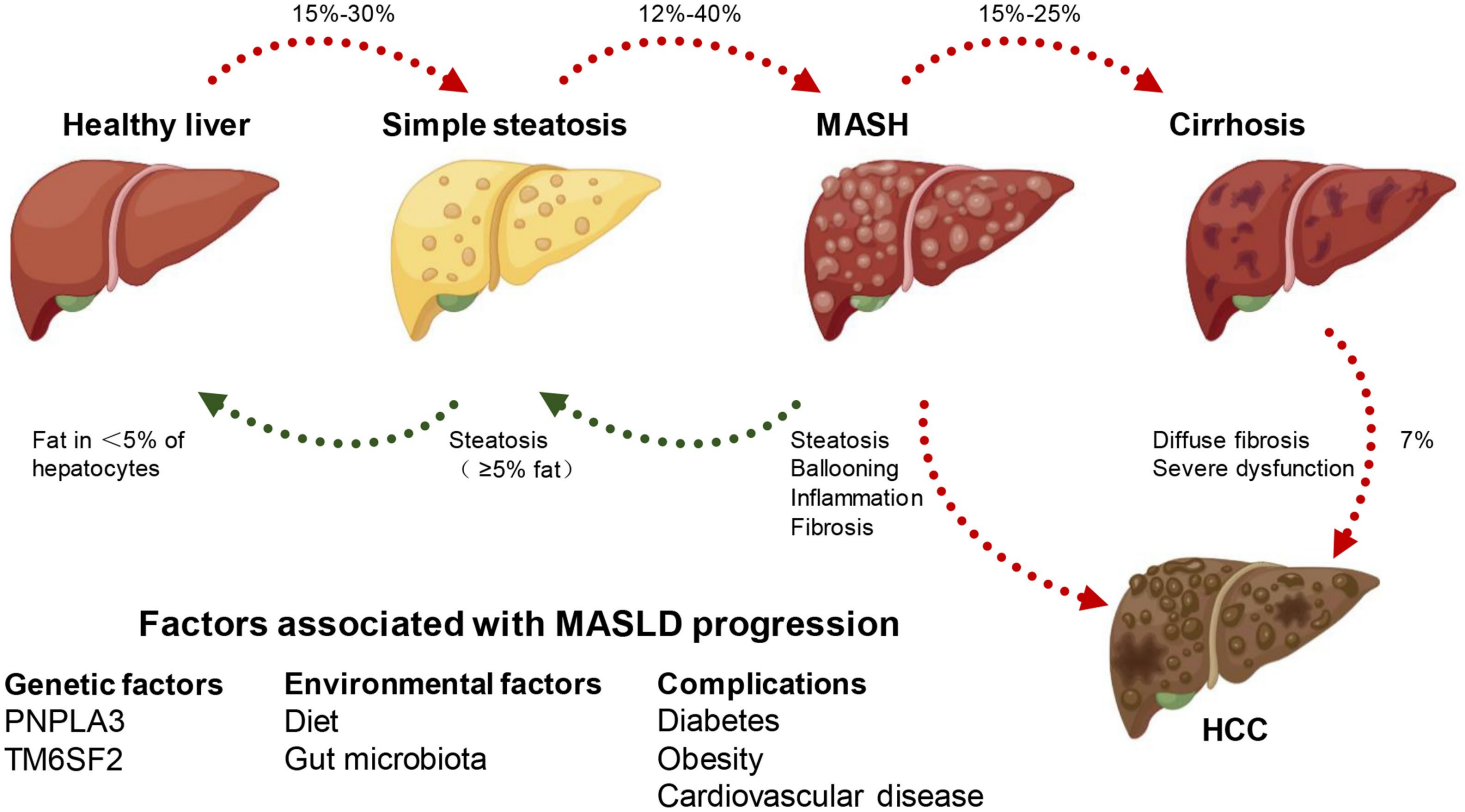
MASLD spectrum. According to different clinical conditions, a healthy liver can develop into simple steatosis and metabolically associated steatohepatitis (MASH), which is still reversible. If MASH develops into cirrhosis, the histopathology becomes irreversible and may develop into hepatocellular carcinoma (HCC). At the same time, genetic, environmental factors and combined diseases can affect the occurrence and development of MASLD.

Excessive fat accumulation in hepatocytes, excluding alcohol and specific liver damage factors, is the defining feature of MASLD, a clinicopathological syndrome caused by the buildup of triglycerides (TG). MASLD comprises various types, initially presenting as simple steatosis, which may progress to MASH. As fat accumulates in the liver, it triggers hepatocyte injury and subsequently activates an inflammatory response. This inflammation involves the infiltration of immune cells into the liver, contributing to the progression of MASH. If left unaddressed, the persistent inflammation can lead to further liver damage, fibrosis, and ultimately the development of more severe conditions like cirrhosis and hepatocellular carcinoma (HCC), resulting in elevated all-cause mortality and liver-related mortality rates (11–13). The disease progression from steatosis to MASH, fibrosis, and HCC is heterogeneous, which occurs after several years or even decades, and is influenced by some unchangeable (such as age, gender, race/nationality, heredity) and changeable factors (including diet, lifestyle, drugs). Hence, the intricate mechanism governing the progression of MASLD remains largely enigmatic. Numerous pieces of evidence indicate that alterations in lipid metabolism in the liver are the primary driving force (14). For instance, the susceptibility to simple steatosis or MASH is heightened by mutations in various genes responsible for governing lipid metabolism, such as patatin-like phospholipase domain 3 (PNPLA3), transmembrane 6 superfamily member 2 (transmembrane 6 superfamily member 2, TM6SF2), farnesyl diphosphate farnesyl transferase 1 (FDFT1), and membrane-bound o-acyl transferase domain containing 7 (MBOAT7) (15) (Figure 1).

**Figure 2:**
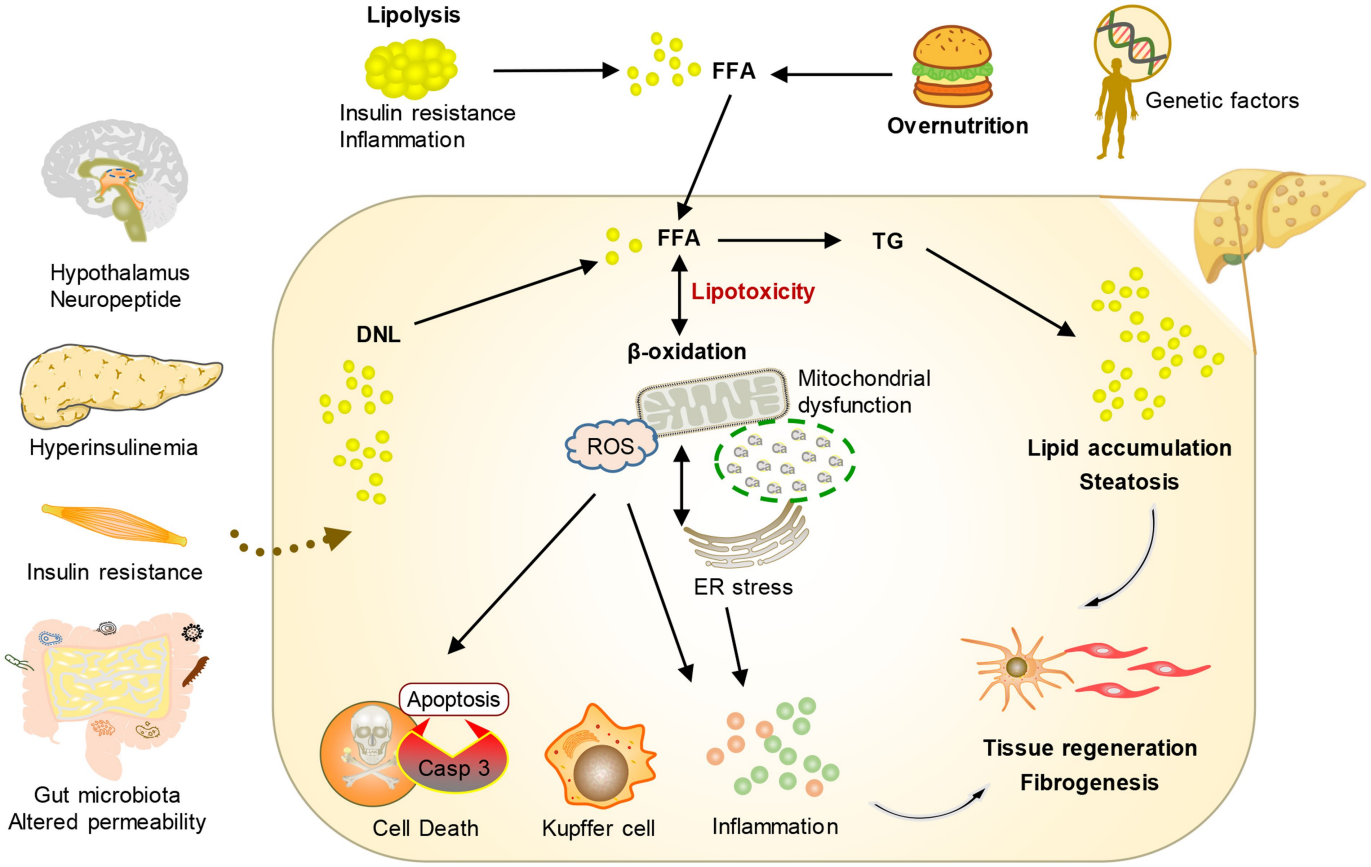
Conceptual diagram of the pathogenesis of MASLD. Under the influence of environmental risk factors and genetic factors, the crosstalk between multiple organs and the liver leads to an increase in fatty acids transported by the liver and the production of new fat. The change in metabolic environment leads to the formation of lipotoxicity, which promotes cell stress and then stimulates inflammation, tissue regeneration, and fibrosis.

Disordered lipid metabolism stands as the fundamental hallmark of MASLD, propelling the advancement of the disease. MASLD develops from an abnormal accumulation of lipids in hepatocytes, which are a type of parenchymal cell in the liver. Steatosis is a histopathological designation denoting the manifestation of lipids in over 5% of hepatocytes (12). Aberrant elevations in intracellular lipids instigate mitochondrial dysfunction and heighten oxidative stress, typically accompanied by augmented *de novo* lipogenesis (DNL) and diminished fatty acid oxidation. Prolonged exposure of hepatocytes to heightened levels of lipid peroxidation and oxidative stress culminates in cell demise orchestrated by lipotoxicity. On the other hand, the macrophages and infiltrating inflammatory cells in the tissue are activated after the damaged liver cells release dangerous signals, which leads to the secretion of pro-inflammatory cytokines, further aggravating the damage and death of liver cells and leading to steatohepatitis (12). In addition, organ cross-talk with the liver and hereditary predisposition can also play an important role in the progression of MASLD (16). Furthermore, in response to this accelerated hepatocyte death, hepatic stellate cells become activated and play a significant role in compensating for the loss of liver tissue (17). Activated hepatic stellate cells undergo a transformation from a quiescent state to a myofibroblast-like phenotype. This activated state enables them to produce and release excessive amounts of extracellular matrix molecules, particularly collagen. The accumulation of collagen and other extracellular matrix components within the liver parenchyma leads to the development of liver fibrosis. Over time, if fibrosis persists, it can progress to advanced stages, ultimately increasing the risk of developing HCC (Figure 2).

**Figure 3:**
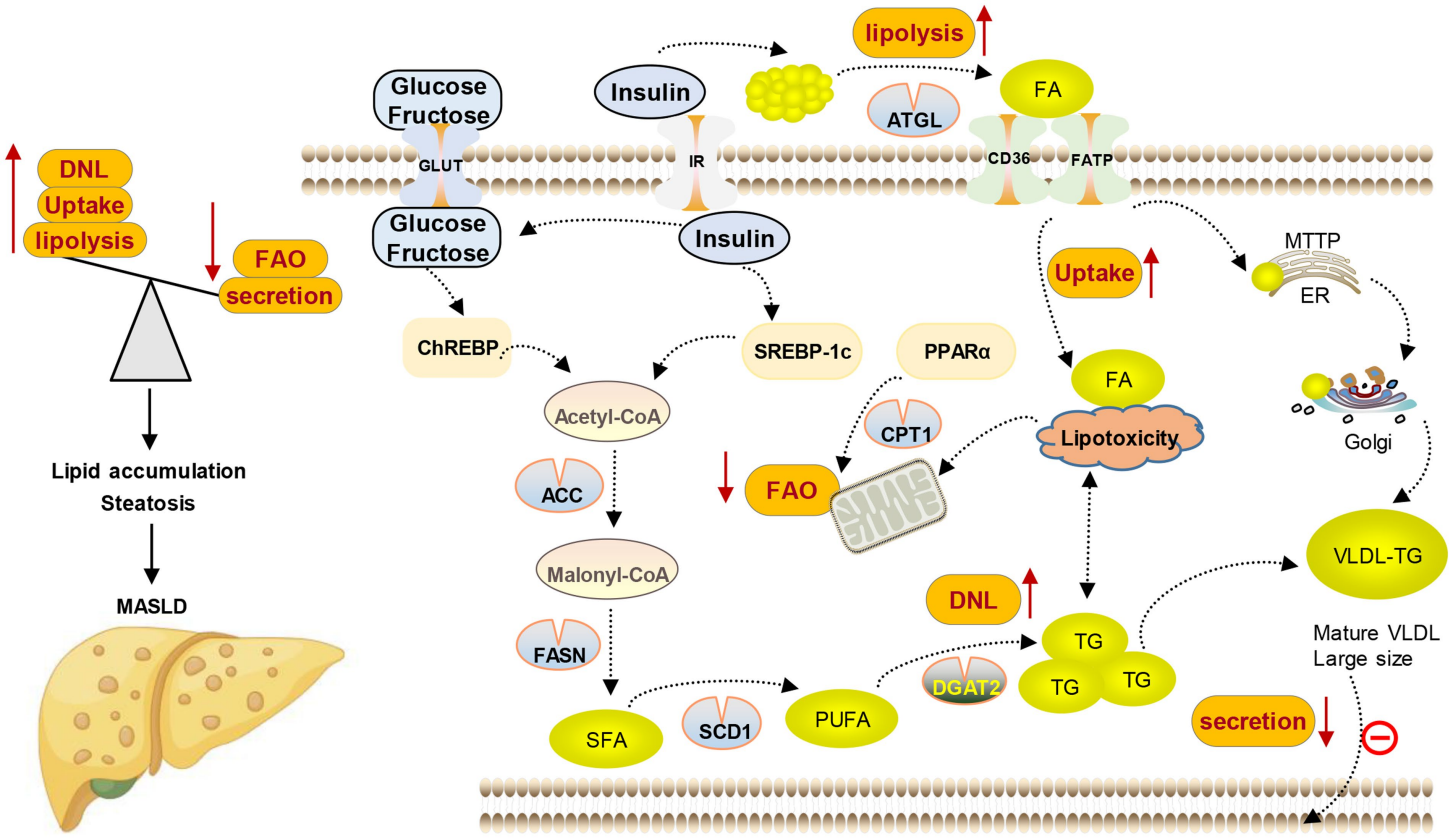
Lipid metabolism disorder in MASLD. Lipid content in the liver is controlled by the balance between lipid input and output, which is mediated by four main pathways. (1) Lipid uptake and transport; (2) *de novo* lipogenesis (DNL); (3) fatty acid oxidation (FAO); and (4) lipoprotein secretion.

MASLD is closely related to various metabolic complications such as obesity, insulin resistance, type 2 diabetes, hyperlipidemia, hypertension, and other cardiovascular diseases due to the significant role of hepatic lipid metabolism in the overall energy balance of the body (18–20). At the same time, studies have demonstrated that the seriousness of MASLD is positively correlated with the heightened possibility of acquiring one or more aspects of metabolic syndrome, indicating that MASLD could be a significant factor in systemic metabolic dysfunction (12, 21, 22). The only reliable and safe approach to managing MASLD lies in the modification of one’s lifestyle, which encompasses weight loss, dietary restrictions, and regular physical activity (23). Currently, no specific pharmacological treatment has been approved for MASLD, either due to limited efficacy or concerns regarding the safety associated with available medications. Therefore, identifying novel therapeutic objectives and creating efficient and secure medication interventions are imperative. The development of MASLD is significantly influenced by the rise in free fatty acids (FFAs) in both blood and hepatocytes. Clinical research is currently focused on reducing the accumulation of fatty acids (FAs) and TG in the liver, as well as reversing or managing the development of simple steatosis and preventing it from advancing to the final stage of MASLD. Hence, this review focuses on the latest advancements in lipid metabolism research, providing a comprehensive overview of the key objectives and pharmaceutical investigations pertaining to lipid metabolism in the context of MASLD (see Figure 3).

## The disturbance of lipid metabolism in MASLD

The liver is the key organ to maintain the energy balance of the human body because it controls the metabolism of different nutrients such as lipids, glucose, and protein. The processing of lipids and FAs from food and adipose tissue by lipid metabolism is crucial in providing a constant supply of energy sources to other organs. During the process of consuming food, lipids from the diet are taken in by the intestine and transported to the liver. In the liver, they undergo metabolism, storage, and circulation throughout the body as TG and cholesterol, which serve as a source of energy for various peripheral tissues. Likewise, when there is limited dietary energy intake during fasting, the liver obtains FAs from adipose tissue to generate ketone bodies. These ketone bodies are then released into the bloodstream and carried to the brain or heart as a substitute for glucose as an energy source (24). Various molecular mechanisms, such as FAs uptake, transport, output, DNL, and fat acid oxidation, are utilized by the liver to regulate FAs metabolism. Altering the equilibrium of these pathways leads to the accumulation of lipids in the liver, resulting in organelle malfunction, cellular injury, apoptosis, inflammation, and persistent activation of fibrosis pathways, all of which exacerbate liver function and advance the development of MASLD (12, 25, 26) (as shown in Figure 2).

### The uptake and transport of FAs increased

A part of the FAs ingested in the liver come from the lipolysis of adipose tissue. Enlargement, contraction, and chronic low-grade inflammation of adipocytes characterize adipose tissue dysfunction in MASLD patients, which can result in increased lipolysis and FFAs production. According to the results of isotope tracing, FFAs from adipose tissue decomposition account for 59% of the accumulation of liver TG (27). Neutral lipolysis is initiated by adipose triacylglycerol lipase (ATGL), which cuts the initial FFAs from TG in lipid droplets, followed by hormone-sensitive lipase, which acts on diacylglycerol, a hydrolysate molecule, releasing two more FAs and one glycerol molecule. The experiment conducted on ABHD15-deficient mice provided evidence for the pathogenic role of FFAs leaked from adipose tissue in the development of MASLD and systemic insulin resistance (28).

FFAs present in the circulation can also be produced from dietary absorption. When we consume dietary fats, they are broken down into FFAs during the process of digestion and absorption in the gastrointestinal tract (29). It’s worth noting that the contribution of dietary absorption to the overall pool of FFAs in circulation depends on factors such as the amount and composition of dietary fats consumed, individual metabolism, and other physiological factors. Some studies have found that the development of MASLD is frequently related to western dietary patterns, particularly the overconsumption of dietary fat (25, 30). Meanwhile, in cases of obesity, the majority of lipids in MASLD originate from the circulating pool of FFAs.

The concentration of FFAs in the plasma influences the rate of fatty acid uptake by hepatocytes. Fatty acid transport proteins (FATPs) and cluster of differentiation 36 (CD36) play key roles in this process. They bind to FFAs present in the plasma and transport them across the hepatocyte membrane. The regulation of FATPs and CD36 expression and activity can be influenced by factors such as hormonal signals (e.g., insulin), dietary composition, and metabolic conditions (31). The dysregulation of these transport proteins can contribute to disruptions in lipid metabolism and the onset of metabolic disorders. Extensive research has been conducted to investigate the role of CD36 in the development of MASLD, as its expression in the liver directly influences the occurrence of hepatic steatosis. A study (32) investigating the involvement of CD36 in lipid metabolism revealed noteworthy findings. In the livers of MASH mice, it was observed that the presence of CD36 on the plasma membrane of hepatocytes, as well as the process of palmitoylation of CD36, exhibited significant augmentation. This, in turn, facilitated the uptake of FFAs by hepatocytes while impeding fatty acid oxidation. Consequently, intracellular lipid accumulation ensued, accompanied by an upsurge in inflammatory responses. Preventing the palmitoylation of CD36 in mice with MASLD can decrease its presence on the hepatocyte plasma membrane and impair its role as a transporter of FFAs. This compelling evidence implies that CD36, along with its regulatory factors, holds promise as a prospective therapeutic avenue for the prevention and treatment of MASLD. In addition, CD36 has the ability to engage with insulin-induced gene-2, enhance the production of sterol regulatory element-binding protein 1c (SREBP1c), stimulate the DNL, and trigger hepatic steatosis (33). FATP isomers 2 and 5 are the primary isomers found in the liver among the six FATP isomers present in mammals. In the mouse model that was fed a high-fat diet, it was found that the removal or reduction of FATP2 and FATP5 genes can decrease the absorption of FAs by hepatocytes, lower the level of TG deposition in the liver, and prevent diet-induced obesity in mice. This suggests that FATP-mediated lipid absorption is a contributing factor to hepatic steatosis (34–36). After FFAs are ingested by the hepatocyte membrane, hydrophobic FAs cannot diffuse freely on the cell membrane, which should be mediated by specific fatty acid binding proteins (FABP) to shuttle between different organelles. FABP1, also known as hepatic FABP or L-FABP, is predominantly expressed in the liver. Its primary function involves facilitating the intracellular transportation of long-chain FAs and regulating FA absorption as well as lipid metabolism. Previous research has indicated that patients with MASLD exhibit a notable rise in FABP1 levels, which can accelerate the progression of the disease and the onset of insulin resistance (37). Emerging research has unveiled that MASLD patients exhibit a notably heightened expression of FABP1 in the intestinal tract. This up-regulation plays a pivotal role in regulating the absorption of dietary FAs and synergistically promotes the initiation of MASH in conjunction with peroxisome proliferator-activated receptor α (PPARα) (38). Consequently, the intracellular uptake and transport of FAs within the liver of MASLD patients are enhanced, leading to the accumulation of detrimental FAs within the hepatic system. This, in turn, fosters the development of fatty degeneration and the progressive advancement of the disease.

### DNL enhancement in MASLD

The conversion of non-fatty acid substrates like glucose, lactate, and amino acids into FAs is known as DNL. DNL relies primarily on glucose as its main carbon source, but fructose can also serve as an additional substrate by bypassing the rate-limiting step of glycolysis catalyzed by phosphofructokinase in the liver (39). The synthetic sources of FAs mainly include endogenous and exogenous FAs. Diet is the primary origin of exogenous FAs, whereas acetyl-CoA, which is discharged during glucose, lipid, and amino acid metabolism, is the fundamental component of endogenous FA synthesis. Acetyl-CoA is converted into malonyl-CoA by acetyl CoA carboxylase (ACC), which is one of the most critical speed-limiting steps in DNL. Malonyl-CoA is the donor of FAs in the process of lipid biosynthesis, which prolongs the FAs chain by two carbon units, inhibits the activity of carnitine palmitoyl transferase 1α (CPT1α), and prevents the β-oxidation process. Within the realm of DNL, several rate-limiting enzymes play prominent roles. ACC and fatty acid synthase (FASN) are involved in the synthesis of FAs, while stearoyl-CoA desaturase-1 (SCD1) significantly contributes to the production of monounsaturated FAs. Additionally, diacylglycerol acyltransferase (DGAT) is involved in the synthesis of triglycerides (TG). Comprehensive isotope tracing investigations have shown that DNL accounts for 26% of FFAs in individuals with MASLD and 10% in those without steatosis (27). Similar studies have further indicated that the contribution of hepatic DNL to intrahepatic TG is even more prominent in obese individuals with MASLD. In contrast, in both obese and lean individuals without MASLD, the contribution of liver TG is 19% and 11%, respectively (40–42). Based on these findings, it is evident that the main difference in nutritional homeostasis between MASLD patients and non-MASLD patients depends largely on the changes in DNL.

The precise mechanism of driving DNL in MASLD is still unknown, but under the background of systemic insulin resistance, due to the increase of circulating insulin and glucose, the activation of two main transcription factors, SREBP-1c and carbohydrate-responsive element binding protein (ChREBP), has been considered a core driving factor (43). In turn, the activation of transcription factors will increase the level of some key enzyme genes in the DNL pathway, including ACC, FASN, and SCD1 (44). The subsequent discussion provides a concise exploration of the roles played by SREBP-1c and ChREBP in MASLD, particularly in relation to DNL.

#### SREBP1c

Through an atypical tyrosine residue in its basic domain named EKRY, SREBP1c has the ability to bind to both sterol regulatory elements and E-box, exhibiting a unique dual DNA binding specificity. SREBPs are immobilized on the endoplasmic reticulum (ER) membrane and translocated into the nucleus after translation to induce the expression of the target gene. In detail, the transportation of the SREBP precursor to the Golgi complex on the ER membrane is facilitated by the SREBP cleavage activating protein (SCAP), which is hindered by the insulin-induced gene (INSIG). The Golgi apparatus cleaves SREBPs using two protein-cleaving enzymes, namely site 1 and site 2 proteases (S1P and S2P). Following the cleavage of proteins, SREBP’s mature version is transported to the nucleus, where it stimulates the activation of lipogenic genes such as FASN and SCD (45). MASLD patients have been confirmed to exhibit elevated levels of SREBP1c expression (46). Selective insulin resistance is also associated with DNL activation induced by SREBP1c, which allows insulin to sustain the elevated DNL level (47). Moreover, SREBP1c plays a direct role in controlling enzymes that are encoded by genes associated with genetic susceptibility to MASLD, including PNPLA3 (48). Simultaneously, the regulation of SREBP1c expression is affected by fat mass and obesity-related genes, as well as its methylation level, indicating that genetic risk factors also play a role in mediating the impact of SREBP1c on MASLD (49).

#### ChREBP

DNL primarily depends on glucose as its main carbon source, and ChREBP plays a role in enhancing fat synthesis in response to glucose. The expression of lipogenesis-related genes is regulated by a complex formed by ChREBP and max-like protein X, which binds to carbohydrate response elements (50). It is reported that there has been an increase in the expression of ChREBP in the liver biopsies of patients with MASH. And a positive correlation exists between the decrease in lipid toxicity and insulin sensitivity and the rise in ChREBP protein content (51). Meanwhile, research has indicated that individuals with liver steatosis exceeding 50% exhibit the greatest levels of ChREBP mRNA expression (52). In ob/ob mice, liver-specific silencing of ChREBP can prevent hepatic steatosis and improve peripheral insulin sensitivity, and its mechanism involves the reduction of hepatic DNL (50, 53). Moreover, the overexpression of ChREBP induced by adenovirus, especially in C57BL/6 J mice fed a high-fat diet, promoted fatty degeneration of the liver (52). Of particular note in the context of MASLD is the fact that fructose consumption is on the rise worldwide, coinciding with the increase in the prevalence of MASLD, and more importantly, fructose is an effective activator of ChREBP (30). Fructose metabolism involves the participation of the liver and small intestine. Glucose transporter 5 is responsible for absorbing and metabolizing the majority (90%) of fructose into glucose and lactic acid in the small intestine, with only a small amount reaching the liver for further processing (54). Preclinical experiments showed that the expression and activity of ChREBP in the liver were improved in mice fed a high fructose diet (39, 55). In the case of excessive fructose, the fructose absorbed by the intestine reaches saturation, and fructose turns into liver metabolism. ChREBP in the liver senses the fructose signal, which is activated by the increase of DNA binding and acetylation (39), which increases DNL and further leads to liver toxicity (56).

In addition, ChREBP plays a direct role in regulating the enzymes encoded by genes carrying genetic risk variations associated with MASLD, such as TM6SF2 (57). TM6SF2 plays a crucial role in the synthesis of very low-density lipoprotein (VLDL) and has been identified as a target of ChREBP in the mouse liver. Moreover, the interaction between the genetic variation of the ChREBP locus and the consumption level of sugar-sweetened beverages (SSB) is related to the plasma concentrations of high-density lipoprotein cholesterol (HDL-C) and TG. Three single nucleotide polymorphisms (SNPs) were found to be positively correlated with the low HDL concentration caused by SSB, and it was found that one of the SNPs might aggravate the high TG concentration caused by SSB (58). Furthermore, it has been observed that in individuals with MASLD, there is an upregulation in the protein expression of the epigenetic regulatory factor known as plant homeodomain finger 2 (PHF2). This factor plays a pivotal role in governing the chromatin assembly of SCD through direct interaction with ChREBP (51). Notably, the expression of host cell factor 1 (HCF-1) is elevated in patients with MASH. HCF-1 binds to the promoter region of lipogenic genes in a ChREBP-dependent manner, thereby creating a necessary condition for the recruitment of PHF2 (59). The regulation of DNL by ChREBP is also influenced by epigenetic factors, as evidenced by the aforementioned research. Thus, the inability to effectively regulate the DNL process constitutes a fundamental characteristic of hepatic lipid accumulation in MASLD patients. The upsurge in DNL serves as a significant mechanism driving the accumulation of TG in individuals with MASLD.

### Damaged fatty acid β-oxidation

The liver possesses a pre-established ability to buffer lipids, allowing it to accumulate surplus FFAs generated by food and adipose tissue and providing a level of defense against lipotoxicity that affects both the local and systemic environment. Mechanisms to counteract lipid toxicity involve enhancing fatty acid β-oxidation and/or converting FFAs into lipids that are metabolically benign and can be securely stored in the liver (60). During fasting or obesity, the circulating FFAs released from adipose tissue increase and reach the liver, which activates PPARα, thus promoting fatty acid β-oxidation. In patients with MASLD, the reduction of fatty acid β-oxidation in the liver is not enough to combat the lipotoxicity caused by toxic lipids (61). For instance, Naguib et al. reported that, compared with healthy individuals, MASLD patients had reduced oxidation of ^13^C-labeled palmitate orally (62). Another study also found that liver fatty acid β-oxidation in patients with MASLD decreased (63). PPARα is the main transcription regulator of liver lipid catabolism and is involved in the regulation of lipid oxidation-related enzymes in the peroxisome, mitochondria, and microsomes (64). In contrast to individuals with simple steatosis, PPARα is down-regulated in patients with MASH, and the expression of PPARα is negatively correlated with the severity of MASLD. A hepatocyte-specific PPARα gene knockout mouse model also shows hepatic steatosis during fasting (65). At the same time, the use of the β3-adrenoceptor agonist CL316243 promotes lipolysis of adipocytes, resulting in an excessive FFA load in the plasma, which cannot be eliminated without sufficient PPARα-mediated fatty acid β-oxidation. Furthermore, PPARβ/δ is highly expressed in the liver, and the lack of PPARβ/δ has been proven to aggravate steatosis in mice (66). Tong’s et al. (67) research found that PPARδ-mediated autophagy activation through the AMPK/mTOR pathway reduced the number of lipid droplets, increased the fatty acid β-oxidation rate, and finally reduced the fatty degeneration of the liver in obese mice. CPT1α, another rate-limiting enzyme in fatty acid β-oxidation, is also confirmed to be down-regulated in MASLD patients (68). CPT1α is an essential transferase for FAs to enter mitochondria, which is also inhibited by malonyl coenzyme A, an intermediate product of DNL. CPT1α is regulated by PPARα and peroxisome proliferator-activated receptor-gamma co-activator α. Recent studies have shown that gene therapy to increase CPT1α activity and fatty acid β-oxidation in the liver has been proven to be effective in reducing steatosis in mice (69). These studies collectively indicate that MASLD patients have decreased fatty acid β-oxidation, and reviving this process could be a viable approach to treating MASLD.

### Decreased lipid transport

The output of water-soluble VLDL particles represents a way to clear excessive liver TG. Although the secretion of VLDL increases in MASLD patients, when the accumulation of liver lipids exceeds 10%, the secretion of VLDL tends to be stable (70). Microsomal triglyceride transfer protein (MTTP) catalyzes the lipidation of apolipoprotein B100 (apoB100) in the ER, leading to the formation of VLDL particles. Evidence indicates that individuals with MASH exhibit a decrease in the production of apoB100 compared to those who are obese or lean without MASH (71). The hepatic gene expression of apoB100, MTTP, and serum levels of VLDL-TG are lower in patients with MASH compared to those without MASH. This suggests that the reduction in VLDL-TG secretion may potentially contribute to the progression of MASLD (72). In addition, the VLDL-TG molecules found in obese individuals are of considerable size, rendering them incapable of penetrating the hepatic vascular sinus and subsequently being discharged into the bloodstream, ultimately resulting in the accumulation of lipids in the liver (70). Increased oxidative stress is another cause of fat accumulation in the liver, as it breaks down apoB100 through the proteasome or nonproteasome and decreases the MTTP transcription level in hepatocytes. Furthermore, the assembly and secretion of VLDL depend on the availability of phosphatidylcholine (PC). Phosphatidyl ethanolamine N-methyltransferase (PEMT) is an enzyme that plays a crucial role in maintaining the availability of PC in the liver. PEMT-mediated synthesis of PC ensures an adequate supply of this lipid for VLDL formation and secretion. Thus, PEMT plays a critical role in maintaining the balance of PC availability in the liver, which in turn affects the synthesis of bile acids and VLDL. Dysregulation or deficiency of PEMT can lead to disruptions in these processes, affecting lipid metabolism and potentially contributing to liver-related disorders such as MASLD and dyslipidemia (73). These events ultimately lead to a decrease in the secretion of VLDL, an increase in lipid accumulation, and the development of hepatic steatosis.

## Medications aimed at regulating lipid metabolism in MASLD

The accumulation of TG in the liver can be promoted by an excess of FFAs in the circulation from either the diet or adipose tissue. FFAs are also produced by DNL. Alternatively, TG can serve as a source of energy or be discharged from the liver in the form of VLDL. The accumulation of TG in the liver, which drives the development and progress of MASLD, is a result of the imbalance between the output (β-oxidation of FFAs and secretion of VLDL-TG) and the input of FFAs (re-esterification of DNL and FFAs from glucose/fructose in the liver). To alleviate the disease burden of MASLD, it would be beneficial to examine the drugs associated with lipid metabolism and molecular mechanisms.

### SREBP1

The ER membrane hosts immature SREBP1 along with SCAP and INSIG, as we have learned from the mature processing of SREBP1. For maturation to occur, SREBP1 must be transported to the Golgi complex along with the capsid protein complex II vesicles. Once there, it is cleaved by S1P and S2P proteases. Several inhibitors have been created to disrupt the processing, maturation, and activity of SREBP1 in light of the aforementioned process, demonstrating that SREBP-controlled lipogenesis is crucial to the advancement of MASH. For example, 25-hydroxysitosterol (25-HL) binds to the INSIG protein, stimulates the interaction between INSIG and SCAP, and keeps them in the ER, thus inhibiting the activation of SREBP and inhibiting lipogenesis (47). 25-HL also shows the dual effects of prevention and treatment, i.e., reducing MASH and atherosclerosis in LDL-R^−/−^ mice induced by diet, reducing the formation of cholesterol crystals, and inhibiting the activation of Kupffer cells. 25-HL exhibits a greater capacity to decrease lipid levels in both the serum and liver compared to lovastatin or obertan cilexetil. Furthermore, it demonstrates favorable safety profiles and pharmacokinetic properties. Fatostatin acts by impeding the exit of SCAP from the ER, inhibiting the weight gain and liver fat accumulation of genetically ob/ob mice (74, 75). Additionally, PF-429242 impedes the functioning of S1P, diminishes the expression of the SREBP target gene within the liver, and attenuates the synthesis rate of cholesterol and fatty acids (76). Furthermore, the boron-containing compound BF175 hinders the transcriptional activity of SREBP1 by impeding the recruitment of SREBP1 to the regulatory complex, consequently reducing the expression of the target gene of SREBP1. And this, in turn, ameliorates the lipid homeostasis of diet-induced obese mice (77).

### ChREBP

Developing drugs targeting ChREBP in MASLD could be an effective approach to addressing the drug treatment of MASLD, given the clinical association between monosaccharide intake (specifically fructose intake) and the rising prevalence of MASLD (78), as well as the significant regulatory function of ChREBP in lipid metabolism.

A new ketohexokinase inhibitor (PF-06835919) has been developed to target the metabolic effects of fructose mediated by ChREBP. By lowering ChREBP activity and DNL, PF-06835919 inhibited ketohexokinase and prevented fructose-induced hepatic steatosis and hyperinsulinemia in preclinical studies using primary rat hepatocytes and a high fructose diet (79). A 2a clinical study showed that PF-06835919 demonstrated favorable safety and tolerance after 16 weeks of treatment for MASH patients and reduced the fat content in the liver (NCT03969719). Another clinical study suggested that PF-06835919 was dose-dependent in the treatment of MASLD. The high-dose PF-06835919 achieved the main clinical end point (the reduction of total liver fat), while the low-dose PF-06835919 (75 mg) did not exhibit any significant therapeutic effect (80). The exploration of the potential therapeutic range of ketohexokinase inhibitors will be extended through continued research on MASH patients (81). Notably, there is evidence that the post-translational modification of ChREBP is mainly mediated by glycosylation. A recent study showed that O-GlcNAc glycosylation increased the stability of ChREBP, promoted the expression of regulated lipogenic target genes, and aggravated hepatic steatosis in a MASLD mouse model (82). Future research on specific drugs should focus on regulating the post-translational modification of ChREBP to fully explore its therapeutic potential in MASLD. In addition, the advantageous impact of MondoA (SBI-993), a ChREBP inhibitor analog, on muscle insulin signaling and systemic glucose tolerance has been demonstrated in the diet-induced obesity mouse model, and whether it has a therapeutic effect in MASLD remains to be further clarified (83).

### PPARs

Numerous efforts have been undertaken to create and examine drugs for the PPAR family in individuals with MASLD. In a phase 2b clinical trial, elafibranor demonstrated positive results as a PPARα/δ dual agonist (84). The main end point was not achieved in a phase III clinical trial for MASH patients (NCT01694849) using elafibranor, resulting in the cessation of further investigation. PPARα/γ can be activated by saroglitaza as a dual agonist. In MASH mice, saroglitazar enhanced liver condition by reducing hepatic steatosis, inflammation, and ballooning and impeding fibrosis progression. Furthermore, it decreased the MASLD disease activity score and liver fat content in the MASH model (85). Saroglitazar (4 mg) was shown to dramatically lower the blood alanine amiotransferase level, insulin resistance, dyslipidemia, liver fat content, indicators of hepatocyte damage, and fibrosis in MASLD patients in a phase II trial (86). In 2020, the Indian Drug Administration approved the listing of saroglitazar magnesium by Zydus Cadila Company for the treatment of MASH patients. At present, a phase II clinical study is under way to determine the therapeutic effect of saroglitazar in the western MASH population (87).

Pemafibrate (K-877), a newly discovered selective PPAR regulator, has been shown in animal studies to ameliorate dyslipidemia, plasma transaminase levels, and the pathological state of MASH (88). Pemafibrate decreases the inflammatory response in the liver of MASLD patients and induces the expression of critical target genes that regulate glucose oxidation and boost fatty acid oxidation (89, 90). At the same time, clinical research indicates that pemafibrate has the ability to decrease plasma triglyceride, VLDL cholesterol, residual cholesterol, and apoC-III levels in a safe and effective manner (91, 92). A previous study showed that, based on magnetic resonance elastography, pemafibrate significantly reduced liver stiffness but did not reduce the fat content of the liver (93). Although pemafibrate has shown gratifying results in reducing triglycerides and cardiovascular risk events (91), adverse events such as chronic kidney disease, acute kidney injury, and venous thromboembolism have been observed in patients (91, 93). Additional investigation is necessary to establish if pemafibrate can mitigate the advancement of MASH and restore histological harm without any apparent negative drug response.

Recent studies suggest that a new generation of broad-spectrum PPAR agonist Lanifibranor (IVA337) shows higher efficacy than single or double PPAR agonists in improving insulin sensitivity, macrophage activation, and reducing liver fibrosis (94, 95). The results of a phase 2b trial comparing lanifibranor 1,200 mg (*n* = 83) or placebo (*n* = 81) for 24 weeks in patients with MASH confirmed by biopsy were published in 2021 (94). Patients who received lanifibranor (1,200 mg) had a 22% higher success rate in reaching the main endpoint compared to the placebo group, as evidenced by a decrease of at least 2 points in their SAF-activity score (steatosis, disease activity, and fibrosis). Lanifibranor (1,200 mg) demonstrated a 48% improvement in at least one fibrosis stage without worsening MASH, compared to 29% with placebo. Meanwhile, in the group receiving lanifibranor treatment, fewer than 10% of patients experienced typical side effects like diarrhea, weight gain, and peripheral edema. An ongoing phase III study (NATiV3) on lanifibranor’s treatment of MASH and F2–F3 fibrosis is expected to show similar therapeutic outcomes.

### ATP-citrate esterase

ATP-citrate esterase (ACLY), a crucial enzyme for lipogenesis, transforms citrate from the tricarboxylic acid cycle in the cytoplasm into acetyl-CoA in the cytoplasm. ACLY plays a crucial role in connecting mitochondrial oxidative phosphorylation with cytoplasmic DNL. The absence of ACLY in hepatocytes can protect against the occurrence of hepatic steatosis and dyslipidemia (96). Hydroxycitric acid isomer (HCA), a derivative of citric acid, was the initial ACLY inhibitor found in sweet potatoes (97). It is reported that the use of HCA interventions can hinder the process of lipogenesis. Sweet potato extract, which contains over 50% HCA, is believed to aid in weight loss (98). This has made sweet potato extract a popular dietary supplement for those looking to lose weight. However, further research and investigation are required to determine the therapeutic impact of HCA on MASLD. ETC-1002, also known as lipoic acid, competitively inhibits ACLY. To become lipoic acid, ACLY must undergo modification by ACSVL1, which is a very long-chain acyl-CoA synthetase. Lipoic acid exhibits a liver-specific effect due to the exclusive expression of ACSVL1 in the liver (96). According to preclinical studies, lipoic acid may reduce abnormal metabolism caused by a high-fat diet and alleviate MASLD by decreasing liver triglyceride and total cholesterol levels, regulating inflammation and fibrosis gene expression, and lowering the MASLD activity score (99). In the diet-induced fatty liver model of female rats, lipoic acid also improves the fatty liver by activating PPARα (100). Recently, a new small molecule, 326E, as an inhibitor of ACLY, showed inhibition of DNL and increased the transport of VLDL-TG, which is expected to become a new choice for MASLD treatment (101).

### ACC

As mentioned above, the conversion of acetyl-CoA to malonyl-CoA mediated by ACC represents a crucial step in DNL. ACC inhibitors can reduce hepatic steatosis, improve insulin sensitivity, and regulate dyslipidemia, making them one of the most promising therapeutic targets for MASLD (102). PF-05221304, a dual inhibitor of ACC1/2 specifically for the liver, has been found to decrease DNL and steatosis in rats fed a western diet. Moreover, it has been shown to reduce inflammation and fibrosis markers in MASH (103). However, it should be noted that PF-05221304 may activate SREBP1c and increase VLDL secretion, potentially leading to hypertriglyceridemia (104). To address this concern, a potential solution is to administer PF-05221304 together with the DGAT2 inhibitor PF-06865571 (105). A study conducted on this combination revealed that, during a 16 weeks follow-up, a single administration of PF-05221304 significantly reduced the accumulation of liver fat and liver damage. However, it also led to a notable increase in serum triglyceride levels, which was observed as early as 2 weeks after administration. Nevertheless, during a subsequent 6-week treatment period, co-administration of PF-06865571 effectively mitigated the hypertriglyceridemia induced by PF-05221304. Furthermore, it greatly reduced the content of liver fat, demonstrating good safety and tolerance.

In addition, arachidonate 12-lipoxygenase (ALOX 12) has the ability to safeguard ACC1 from lysosomal deterioration and facilitate the development of MASH (106). A recent investigation unveiled a novel approach to targeting ALOX12-ACC1. The compound IMA-1 demonstrated promising potential in the treatment of MASLD by stimulating protein degradation and modulating the activity of ACC1. However, it did not yield a substantial reduction in polyunsaturated fatty acids or an elevation in circulating triglyceride levels (107).

### FASN

Cerulenin was first discovered as an antifungal drug in 1976, when it was found that it could inhibit the activity of FASN (108). To specifically inhibit FASN, a synthetic analog called C75 was developed due to the fact that it also inhibits the biosynthesis of sterols by inhibiting β-hydroxy-β-methylglutaryl-CoA synthetase, as cerulenin does (109). The administration of cerulenin and C75 resulted in a decline in food intake and weight in mice (110, 111). This effect is likely attributed to the elevation of malonyl-CoA levels within the hypothalamus, consequently impeding the appetite signal of the hypothalamus that is typically induced by fasting (112). Nonetheless, the specific mechanism through which cerulenin and its derivative C75 reduce food consumption remains uncertain. An inhibitor of FASN that can be taken orally is TVB-2640. Phase I clinical trials on obese male individuals showed that TVB-2640 inhibited the DNL and reduced the plasma level of lipids (113). In a phase 2a clinical trial (NCT03938246), TVB-2640 exhibited noteworthy outcomes. Over a period of 12 weeks, TVB-2640 demonstrated a substantial reduction in liver fat content and displayed dose-dependent improvements in biomarker levels related to biochemistry, inflammation, and fibrosis in patients with MASH. Additionally, another phase 2 clinical study for MASH patients is currently underway (NCT04906421). FT-4101, a potent and selective compound with oral availability, has emerged as another promising candidate for inhibiting FASN function by precisely targeting specific regions. Its potential efficacy in the treatment of MASH is currently being investigated. In two parallel studies, FT-4101 demonstrated dose-dependent inhibition of DNL, and a dosage of 3 mg of FT-4101 exhibited a significant reduction in hepatic steatosis over a 12 weeks period, similar to monotherapy (114). These clinical trials collectively underscore the considerable therapeutic potential of targeting FASN as a treatment approach for MASLD.

### SCD1

The activity of SCD1 increased in simple steatosis and MASH patients (115), while obese mice with SCD1 gene knockout in the liver induced by diet showed a decrease in steatosis and insulin resistance (116). 3β-aramcocholic acid (aramchol) is an oral fatty acid-bile acid conjugate that can partially inhibit the level of the SCD1 protein in the liver and reduce fibrosis in animal models of MASLD (117). In a 12-week phase 2a clinical trial, compared with placebo, 300 mg of aramchol per day significantly reduced liver fat content in a dose-dependent manner (118). Larger doses of aramchol (400 mg and 600 mg) also showed good therapeutic effects in another 2b clinical trial (triglyceride content in the liver as the main end point), and the number of serious adverse events was lower than 5% (117). Based on the efficacy, safety, and tolerability of aramchol administration, a phase 3 clinical trial (NCT04104321) for MASH patients is currently under way.

### DGAT2

DGAT2 serves as the catalyst for the last step in hepatic triglyceride synthesis. By reducing DGAT2 production, it is possible to enhance steatosis, serum lipid levels, and triglyceride biosynthesis. A randomized controlled trial (NCT04932512) is currently investigating the potential of improving MASH through the use of magnetic resonance imaging-derived proton density fat fraction after a 49 weeks period without exacerbating fibrosis and hepatic steatosis. As previously mentioned, the combination of the DGAT2 inhibitor PF-06865571 and the ACC1/2 inhibitor PF-05221304 can obviously reduce hypertriglyceridemia caused by PF-05221304 alone.

### ATGL

A potential therapeutic strategy for MASLD involves reducing the mobilization of FAs and their corresponding plasma concentrations by inhibiting the lipolysis of adipose tissue. The specific inhibition of ATGL using the chemical inhibitor atglistatin has shown promising results in mice, effectively reducing lipolysis, lipid deposition, weight gain, and insulin resistance induced by a high-fat diet (119). It is important to note that this inhibition does not lead to lipid accumulation in ectopic tissues such as skeletal muscle or the heart, even with prolonged treatment. Thus, mice treated with atglistatin do not experience the severe heart steatosis and cardiomyopathy observed in the genetic mouse model lacking ATGL. These findings indicated that pharmacologically inhibiting ATGL could be a viable therapeutic approach for MASLD.

**Figure 4:**
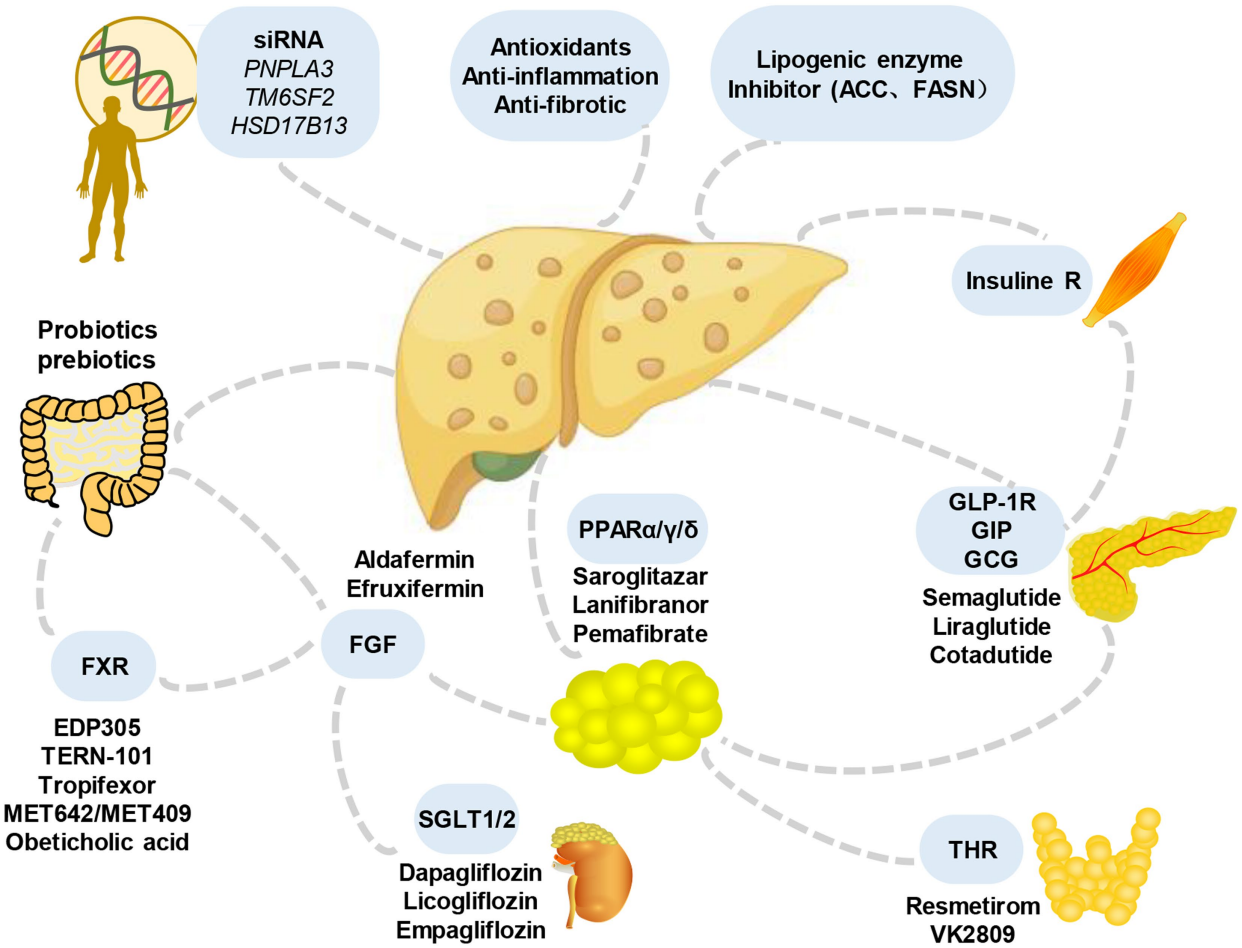
Main candidate drugs for the treatment of MASLD. Numerous prospective treatments for MASLD and its advanced variants have received extensive research over the past years, throwing some light but also raising some questions. They include some medications for lowering blood sugar (such as GLP-1 receptor agonists and SGLT-2 inhibitors), antioxidants (like vitamin E), statins or other lipid-lowering medications, bile and non-bile acid FXR agonists, and others. FGF, fibroblast growth factors; SGLT, sodium-glucose cotransporter; THR, thyroid hormone receptor; GLP-1R, glucagon-like peptide-1 receptor; GIP, gastric inhibitory peptide; GCG, glucagon; FXR, farnesoid X receptor.

In addition, considering that various signals contributing to MASLD originate from outside the liver, including the intestine, adipose tissue, and endocrine system, interorgan communication plays a significant role in the occurrence and development of the condition (120). Therefore, the treatment of extrahepatic mediators is also under evaluation (Figure 4). For example, resmetirom, a thyroxine receptor beta agonist, has emerged as a pioneering medication in achieving significant hepatic histologic endpoints in a clinical trial (NCT03900429). The analysis showed that resmetirom met two primary endpoints and exhibited potentially clinically significant effects compared to placebo at both daily oral doses of 80 mg and 100 mg. These effects encompassed MASH symptom remission, a ≥2-point reduction in non-alcoholic fatty liver activity score (NAS) without worsening liver fibrosis, at least one stage of improvement in liver fibrosis without worsening NAS, and a reduction in the key secondary endpoint of LDL-C that significantly exceeded that of the placebo group. Furthermore, noteworthy attention should be given to single nucleotide polymorphisms (SNPs), such as PNPLA3 and TM6SF2, which are intricately linked to the onset and progression of MASLD. Understanding the impact of these SNPs can provide valuable insights into the underlying mechanisms and potential therapeutic targets for the disease.

## Conclusion and perspectives

Over the last few decades, significant advancements have been made in comprehending the pathogenesis of MASLD. Excessive energy substrates, especially carbohydrates, cause DNL in the liver, while fat in the diet and adipose tissue with metabolic disorders provide excessive FFAs. Although the initial storage of liver triglycerides may play a buffering role, the production of toxic lipid metabolites is the main feature of progressive MASLD, which involves the production of cell pressure, mitochondrial dysfunction, the increase of reactive oxygen species, and the development of endoplasmic reticulum pressure (Figure 2). Reducing liver lipid deposition by targeting different pathways, especially key molecules, is of great clinical value to help identify key metabolic weaknesses that can be targeted to attenuate hepatic damage, inflammation, and fibrosis. This review systematically introduces the causes of liver lipid metabolism imbalance and the drug development and research progress of key molecules, aiming at a deeper understanding of the role of lipid metabolism in MASLD and providing ideas for clinical drug development from the molecular mechanism.

As we delve deeper into our research, it is crucial to consider the impact of ethnic and individual variances. By integrating genetic and metabolic factors, a more comprehensive understanding of the pathogenesis of MASLD can be achieved, and individualized therapeutic strategies can be developed. This may include explorations in genomic studies, metabolomic analysis, and drug development to accelerate therapeutic advances in MASLD. The application of this holistic approach is expected to provide more effective treatment options for patients with MASLD and improve the feasibility and success of treatment.

Additionally, due to the intricate nature of the pathophysiology involved in MASLD, it is possible that a solitary medication may not suffice to reverse the disease, necessitating the use of a combination therapy. The combination therapy of two or more drugs may improve the curative effect through complementation or synergy and improve tolerance by using a lower dose of candidate drugs. However, it is still a challenge to determine the ideal combination of drugs. The ideal combination therapy will aim at multiple steps of the pathogenesis, from energy balance to fibrosis. In addition to liver-oriented therapy, it should also include drugs with significant metabolic effects (such as drugs for treating type 2 diabetes). In a randomized phase II trial (NCT03987074), the use of a combination therapy consisting of semaglutide, farnesoid X receptor agonist cilofexor, and/or ACC inhibitor firsocostat was found to be more effective in improving liver steatosis, biochemistry biomarkers, and fibrosis compared to using a single drug therapy. Another example is the pairing of cilofexor and firsocostat, which improves various measures of MASH activity such as ballooning, inflammation, and steatosis and potentially exhibits anti-fibrosis properties (NCT03449446). Hence, by identifying and refining the most effective combinations, researchers can improve treatment outcomes and address the various aspects of the disease. Overall, it is anticipated that in the coming years, obstacles to the prevention, diagnosis, and treatment of MASLD will be surmounted.

## Author contributions

XP: Writing – original draft, Writing – review & editing. KA: Software, Writing – original draft. HH: Writing – original draft. XF: Supervision, Writing – original draft. SL: Supervision, Writing – original draft. ZA: Conceptualization, Supervision, Validation, Writing – original draft. GR: Investigation, Writing-original draft, Software, Writing - review and editing. XL: Writing-original draft, Writing - review and editing.
